# First person – Melissa Hinman

**DOI:** 10.1242/dmm.049118

**Published:** 2021-06-14

## Abstract

First Person is a series of interviews with the first authors of a selection of papers published in Disease Models & Mechanisms, helping early-career researchers promote themselves alongside their papers. Melissa Hinman is first author on ‘ Zebrafish *mbnl* mutants model physical and molecular phenotypes of myotonic dystrophy’, published in DMM. Melissa is a postdoctoral fellow in the lab of Dr Karen Guillemin at the University of Oregon, Eugene, OR, USA, using zebrafish models to better understand the mechanisms underlying the phenotypes of the genetic disorder myotonic dystrophy and to develop potential therapeutics.


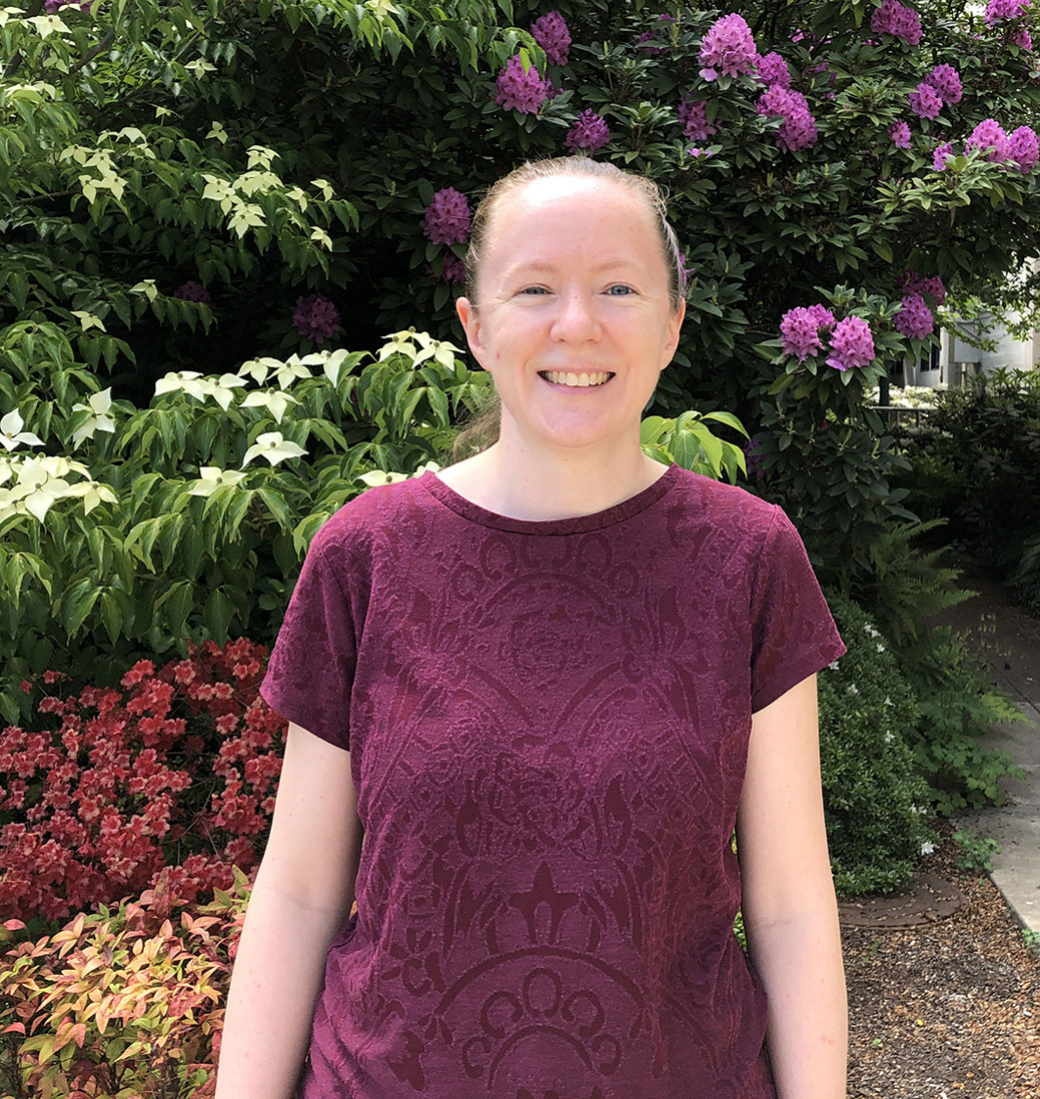


**Melissa Hinmans**

**How would you explain the main findings of your paper to non-scientific family and friends?**

Myotonic dystrophy (DM) is a genetic disorder that is most well known for causing muscle symptoms, but also affects many other parts of the body, such as the brain, heart and digestive system. Individuals with DM have an extra-long piece of RNA within their cells that acts like a sponge, sucking up the MBNL family of proteins and preventing them from performing their normal functions. We mimicked DM in zebrafish by breaking the genes that are used to produce these MBNL proteins. We found that DM-model zebrafish had many disease-relevant symptoms, including decreased body size, impaired movement, and many of the same changes that are observed on a molecular level in DM patients. These fish will be useful for future studies to understand the mechanisms underlying DM symptoms and for testing potential treatments.

“I was surprised to find that triple homozygous *mbnl* mutant zebrafish were viable to adulthood, when analogous mouse models were not.”

**What are the potential implications of these results for your field of research?**

We have created the first complete panel of zebrafish single, double and triple homozygous *mbnl* mutants, and analysed genome-wide alternative splicing changes within all of them. These fish models will be an invaluable toolset for studying how individual Mbnl proteins, and the alternative splicing changes associated with them, contribute to disease-relevant phenotypes. The *mbnl* mutant fish, as well as stable transgenic CTG-repeat-expressing fish that are currently in development, also have great potential for large-scale testing of therapeutic compounds.

**Figure:**
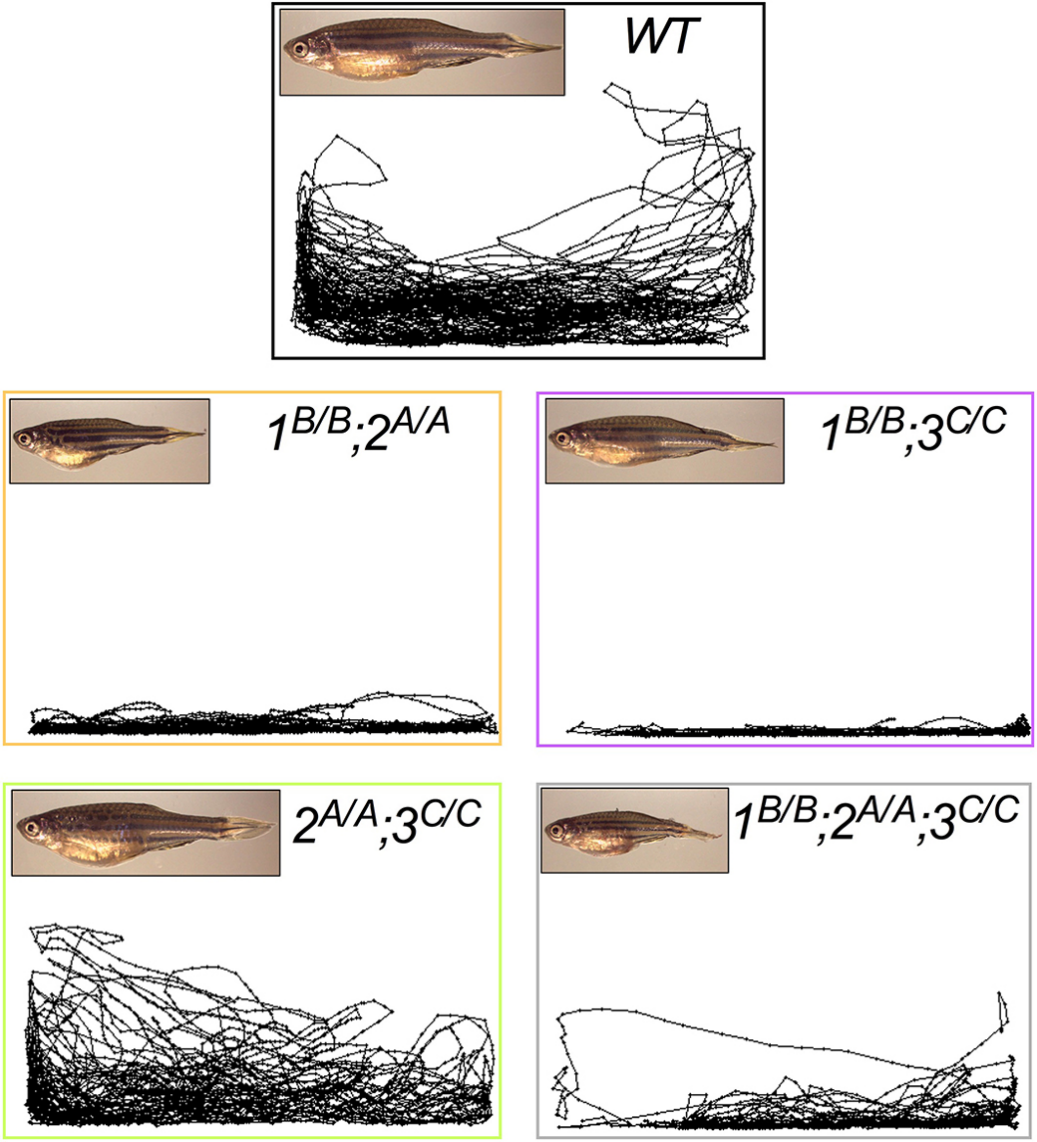
**Loss of Mbnl function led to DM-relevant phenotypes including reduced size and impaired movement.** Shown are representative images of compound *mbnl* mutant fish and traces of their altered swimming patterns.

**What are the main advantages and drawbacks of the model system you have used as it relates to the disease you are investigating?**

One major advantage of using zebrafish to study myotonic dystrophy is that many internal phenotypes can be studied directly in transparent larval fish, which would be difficult or impossible to do using other model systems such as the mouse. We are currently exploiting this fact to study DM-relevant digestive phenotypes. Another advantage is that zebrafish can be generated in large numbers, which is valuable for studying subtle or variable phenotypes and for testing potential therapeutics.

A disadvantage to using zebrafish to study DM is that they are more distantly related to humans than mice. Although zebrafish exhibited many of the same alternative splicing changes that are observed in individuals with DM, not all were conserved. In addition, *mbnl* mutant fish did not exhibit some of the muscle histology changes that were observed in humans and DM-model mice, suggesting that they may not be an appropriate model system for studying some muscle phenotypes.

**What has surprised you the most while conducting your research?**

I was surprised to find that triple homozygous *mbnl* mutant zebrafish were viable to adulthood, when analogous mouse models were not. I am interested in understanding why this is true. Did our *mbnl* mutations lead to an incomplete loss of function? Does something about zebrafish physiology eliminate the need for Mbnl proteins to survive? Are certain alternative splicing changes that cause lethality in mouse compound *mbnl* mutants not conserved in zebrafish?

**Describe what you think is the most significant challenge impacting your research at this time and how will this be addressed over the next 10 years?**

I think a big challenge in the DM field is understanding how individual tissues contribute to overall disease phenotypes. There is great potential to develop tissue-specific DM models in zebrafish. In addition, as single-cell sequencing technology improves in the coming years, it may be possible to analyse all alternative splicing changes in every tissue of DM model larval zebrafish at once, giving a more complete picture of how tissue-specific molecular changes contribute to disease phenotypes.

“[…] a big challenge in the DM field is understanding how individual tissues contribute to overall disease phenotypes.”

**What changes do you think could improve the professional lives of early-career scientists?**

In brief moments of despair, it feels a bit like science is an elaborate pyramid scheme in which each PI recruits multiple grad students and postdocs to work under them with the promise that they, too, can become PIs, when that can't possibly be true for all of them. I think that more emphasis should be placed on the diverse career opportunities available to trainees, and that we should reduce the stigma associated with pursuing any career outside of tenure-track academia.

**What's next for you?**

We recently discovered intestinal phenotypes in the *mbnl* mutant fish that were developed in this study and I'm currently investigating the mechanisms underlying these phenotypes. This is an exciting development for the DM field, as DM-related digestive phenotypes are poorly understood and other animal model systems are not ideal for studying them. I'm also characterizing newly developed fish that model DM through a complementary approach, the stable overexpression of CUG-repeat RNA. Longer term, I would like to use these zebrafish models to investigate new therapies for DM, working either in academia or industry.
